# Allosteric regulation in NMDA receptors revealed by the genetically encoded photo-cross-linkers

**DOI:** 10.1038/srep34751

**Published:** 2016-10-07

**Authors:** Meilin Tian, Shixin Ye

**Affiliations:** 1 Shanghai Key Laboratory of Brain Functional Genomics, East China Normal University, Shanghai, China; 2 Ecole Normale Supérieure, Institut de Biologie de l’Ecole Normale Supérieure (IBENS), Paris, France; 3 Institut National de la Santé et de la Recherche Médicale, U1024, Paris, France; 4 Centre National de la Recherche Scientifique, UMR 8197, Paris, France

## Abstract

Allostery is essential to neuronal receptor function, but its transient nature poses a challenge for characterization. The N-terminal domains (NTDs) distinct from ligand binding domains are a major locus for allosteric regulation of NMDA receptors (NMDARs), where different modulatory binding sites have been observed. The inhibitor ifenprodil, and related phenylethanoamine compounds specifically targeting GluN1/GluN2B NMDARs have neuroprotective activity. However, whether they use differential structural pathways than the endogenous inhibitor Zn^2+^ for regulation is unknown. We applied genetically encoded unnatural amino acids (Uaas) and monitored the functional changes in living cells with photo-cross-linkers specifically incorporated at the ifenprodil binding interface between GluN1 and GluN2B subunits. We report constraining the NTD domain movement, by a light induced crosslinking bond that introduces minimal perturbation to the ligand binding, specifically impedes the transduction of ifenprodil but not Zn^2+^ inhibition. Subtle distance changes reveal interfacial flexibility and NTD rearrangements in the presence of modulators. Our results present a much richer dynamic picture of allostery than conventional approaches targeting the same interface, and highlight key residues that determine functional and subtype specificity of NMDARs. The light-sensitive mutant neuronal receptors provide complementary tools to the photo-switchable ligands for opto-neuropharmacology.

Ligand-gated ion channels (LGICs) in response to the binding of chemical messengers, such as neurotransmitters, mediate synaptic transmissions. LGICs are membrane proteins composed of multiple subunits. Allosteric modulators selectively binding at sites distinct from agonists binding domains can affect channel function. Understanding the mechanisms of allosteric regulation and their impacts in gating is an important goal in neurophysiology and neuropharmacology. Recently, the electron microscopic structures and high-resolution crystal structures of several full-length LGICs^1^,^2^,^3^ provided valuable insights into the interaction surface between different subunits. However, the diversity of the multi-subunit complexes and the transient nature of allosteric dynamics impose challenge to probe.

A functional hallmark of NMDARs is that allosteric regulations by binding of small molecules at the interface of the N-terminal domains (NTDs)^4^,^5^, which lay most distal to the pore region of the receptors, are subunit specific. NMDARs belong to the ionotropic glutamate receptors (iGluR) family mediating excitatory synaptic transmissions associated with learning and memory^6^,^7^. Functional NMDARs require at least two different subunits to assemble as a tetrameric complex, commonly consisting of two GluN1 and two GluN2 (A-D) subunits. Ifenprodil and Zn^2+^ are two kinds of subunit-selective allosteric inhibitors that bind at distinct sites of NTDs of NMDARs. Although much information has been gained on the functional roles of ifenprodil and Zn^2+^ in the NMDARs allosteric signaling, whether and how they use differential structural pathways for inhibition remains unclear. Synthetic compounds ifenprodil and related phenylethanolamine derivatives, which specifically inhibit GluN1/GluN2B receptors, have been intensely studied for their potential use in the treatment of various neurological disorders and diseases^7^. Crystal structures demonstrate that ifenprodil and derivatives bind at the NTD dimer interface between the GluN1/GluN2B^8^,^9^,^10^. It is distinct from the Zn^2+^ binding site which is in the GluN2B-NTD cleft revealed by the isolated GluN2B NTD structure^11^,^12^. Zn^2+^ acting as an endogenous allosteric modulator plays a key role in physiology by shaping NMDAR synaptic currents and in pathology by modulating pain processing^7^,^13^. It targets both GluN1/GluN2A and GluN1/GluN2B receptors with higher affinity for GluN2A than GluN2B containing receptors.

To differentiate the mechanisms between Zn^2+^ and ifenprodil induced allosteric inhibition, we set out to apply the photo-chemical approach established previously^14^. It combines genetically encoded light-sensitive unnatural amino acids (Uaa)^15^,^16^ at the targeted receptors with simultaneous electrophysiological analysis to identify structural elements associated with specific allosteric modulation. With this approach, the photo-cross-linker *p*-azido-L-phenylalanine (AzF) is introduced at a specific position in the receptor. Functional changes can be detected if the light-stimulated crosslinking causes structural rearrangements. The distance-dependent crosslinking may provide information for interfacial contacts. Previously we have applied the strategy at the Zn^2+ ^17 and ifenprodil^14^ binding sites using well-studied sites to establish the proof-of-concept. However, given the lack of direct proof of cross-linking, the conformational changes associated and hence their differential mechanisms of modulation are unclear. Here we target the ifenprodil interface by systematically introducing AzF at the GluN2 subunit and identified a novel allosteric potentiation mechanism in the GluN2B containing receptors, which is completely different from previous reported work in GluN1 mutant with the light inhibition phenotype and crosslinking mechanism^14^. Furthermore, using the light-sensitivity of the mutant, we have differentiated the inhibition mechanisms between Zn^2+^ and ifenprodil binding. In this case, AzF as a light-sensitive crosslinking probe is decisively advantageous to reveal the specific functional role GluN1/GluN2B interface in the transduction of ifenprodil inhibition, which conventional approaches failed to reveal. These findings pave the way for guiding the design of subtype-specific compounds with therapeutic value for neurological disorders and diseases. We further discuss the potential applications of the identified light-sensitive NMDARs in the context of opto-neurophamarcology.

## Results

### Generating AzF mutants in GluN2 subunits

**Figure 1:**
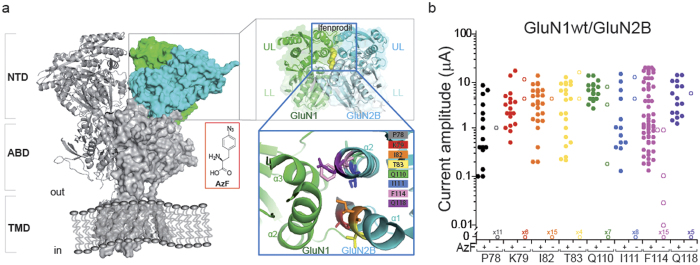
Incorporation of AzF at the NTD dimer interface. (**a**) Side view of GluN1/GluN2B receptor crystal structure with ifenprodil molecule omitted for clarity (PDB 4PE5). An intact receptor forms a tetrameric complex assembled as a dimer of dimers. There are three major domains: N-terminal domain (NTD) that harbors several sites for allosteric modulators; agonist binding domain (ABD) that binds glycine (or D-serine) in GluN1 and glutamate in GluN2 subunits; and transmembrane domain (TMD) that comprises the ion-channel pore. These three domains—NTD, ABD, and TMD are arranged in layers. One NTD dimer in complex with ifenprodil (yellow sphere) is highlighted. Helices from the upper-lobe upper-lobe (UL-UL) interface (helix α2 & α3 of GluN1, and helix α1 & α2 of GluN2B) are enlarged. Residues in the GluN2B subjected to amber mutation are represented as colored sticks: P78 (grey), K79 (red), I82 (orange), T83 (yellow), Q110 (green), I111 (blue), F114 (pink), Q119 (purple). (**b**) Currents measured from oocytes co-injected with four plasmids: GluN2B amber mutant, wt GluN1, Yam and AzF-RS. For each injection, oocytes were spitted into two batches: one batch was incubated in the medium with 1 mM AzF. For each condition, 6–52 oocytes were tested, currents >10 nA were plotted; the other batch was incubated in the absence of AzF. At least 5 oocytes in each condition were tested.

To identify the functional role of ifenprodil binding interface, we engineered AzF mutants using the ifenprodil-bound GluN1/GluN2B full-length structure as a guide. The NTDs have bi-lobed clamshell-like architectures composed of upper-lobe (UL) and lower-lobe (LL) domains. In both ifenprodil-bound and R_o_25-6981-bound (phenylethanoamine derivative) forms of the structures (Fig. 1a)^8^,^9^, the ifenprodil binding site is clearly identified at the upper-lobe upper-lobe (UL-UL) interface. Ifenprodil makes direct interactions with the UL-UL mainly through hydrophobic interactions between the benzylpiperidine group and a cluster of aliphatic and aromatic residues from two helices (α2 and α3) of the GluN1 and the two helices (α1 and α2) from GluN2B. We selected eight sites along these helices of the GluN2 subunit as candidates for AzF incorporation based on the following criteria: 1) the site should reside in the interface to enable light-induced cross-linking between the two subunits; 2) distances between the C_α_s of GluN2B and closest GluN1 residues shall fall in the range of 6–11 Å. In this way eight sites (α1 helix: P78, K79, I82, T83; α2 helix: Q110, I111, F114, Q118) were chosen (Fig. 1a). In order to compare with the GluN1/GluN2A receptors that lack X-ray crystal structures, we also generated amber mutations in GluN2A subunit (α1 helix: P79, K80, I83, T84; α2 helix: Q111, M112, F115, Q119). Based on primary sequence comparisons between GluN2A and GluN2B, all sites involved in ifenprodil binding in GluN2B subunit are conserved in GluN2A except GluN2A-M112 (GluN2B-I111).

We co-injected into *Xenopus laevis* oocytes four DNA plasmids containing genes encoding the AzF aminoacyl tRNA-synthetase (AzF-RS), the orthogonal suppressor tRNA (Yam), the wild-type GluN1, and the GluN2 subunit with an amber stop codon mutation at the desired position^17^. The oocytes were maintained in the external medium supplemented with 1mM AzF. The expression of AzF mutants was verified by two-electrode voltage-clamp (TEVC) recordings. Robust NMDAR-mediated currents were observed 2–3 days after co-injection of the four plasmids (Fig. 1b, Supplementary Fig. 1a,c). More than 50% of oocytes showed expression. All AzF mutants were produced as functional receptors, with maximal agonist induced current amplitude values in the range of 0.1–10 μA (variation is caused by DNA injection into different batches of oocytes), suggesting that NMDARs tolerate AzF at the chosen site despite being buried at the interface. In the absence of AzF, the majority of oocytes generated no significant signals due to premature termination of protein synthesis, confirming the engineered aaRS/suppressor tRNA pairs derived from different species do not cross-react with endogenous tRNA or aaRS in the host cellular systems^18^,^19^. In a small fraction of oocytes (<5%), NMDARs were expressed without AzF provided in the media (amber suppression leakage), indicating that AzF-RS has a low level of activity using endogenous amino acids (likely tyrosine) as substrate to aminoacylate the suppressor tRNA^17^ (Fig. 1b, Supplementary Fig. 1b). Overall, these results demonstrated that all the tested sites tolerated AzF mutation and led to efficient expressions of full-length NMDARs in response to the aaRS/suppressor tRNA amber rescue.

### Screening of AzF mutants for UV-induced functional changes

**Figure 2:**
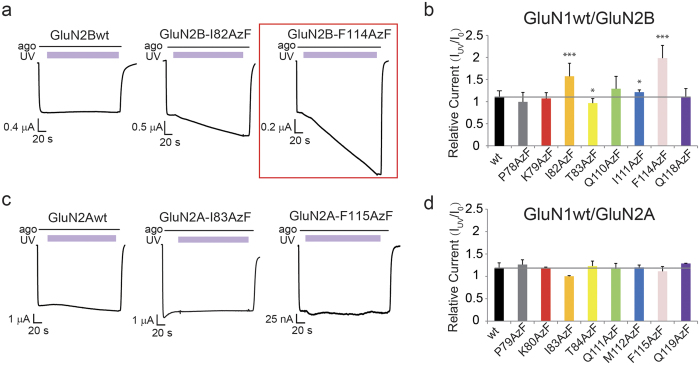
UV sensitivity of AzF mutant receptors. (**a**) Representative current traces measured from oocytes expressing wt and AzF mutant receptors during UV illumination. Two GluN2B mutants (GluN1/GluN2B-I82AzF and GluN1/GluN2B-F114AzF) showed UV-induced potentiation. (**b**) Relative currents (I_uv_/I_o_) measured on oocytes expressing GluN2B receptors of AzF mutants: GluN2Bwt (1.08 ± 0.12, n = 26), GluN2B-P78AzF (0.99 ± 0.21, n = 4), GluN2B-K79AzF (1.07 ± 0.13, n = 4), GluN2B-I82AzF (1.57 ± 0.30, n = 5), GluN2B-T83AzF (0.97 ± 0.10, n = 9), GluN2B-Q110AzF (1.29 ± 0.28, n = 3), GluN2B-I111AzF (1.21 ± 0.05, n = 2), GluN2B-F114AzF (1.97 ± 0.29, n = 21), GluN2B-Q118AzF (1.11 ± 0.18, n = 5). (**c**) AzF mutations in homologous sites in GluN2A and GluN2A wild-type receptors had no UV sensitivity. (**d**) Relative currents (I_uv_/I_o_) measured on oocytes expressing GluN2A receptors of AzF mutants: GluN2A wt (1.18 ± 0.12, n = 11), GluN2A-P79AzF (1.26 ± 0.11, n = 3), GluN2A-K80AzF (1.17 ± 0.03, n = 3), GluN2A-I83AzF (1.00 ± 0.02, n = 2), GluN2A-T84AzF (1.23 ± 0.11, n = 13), GluN2A-Q111AzF (1.17 ± 0.12, n = 4), GluN2A-M112AzF (1.20 ±  ± 0.05, n = 2), GluN2A-F115AzF (1.11 ± 0.11, n = 7), GluN2A-Q119AzF (1.29 ± 0.003, n = 2).

To evaluate whether AzF photo-activation in GluN2B produces functional changes, we applied a time-resolved assay^10^ by combining online application of UV light and simultaneously recording of agonist-induced currents using TEVC. Current after UV illumination (I_uv_) were compared to currents before UV illumination (I_o_) and yielded the relative current changes (I_uv_/I_o_). Among eight maximally activated GluN1/GluN2B-AzF mutants, two mutants (GluN2B-I82AzF and GluN2B-F114AzF) showed robust UV induced potentiation (Fig. 2a) (I82AzF: 1.57 ± 0.30, n = 5 and F114AzF: 1.97 ± 0.29, n = 21). The other six mutations had only small or no effects: GluN2B-P78AzF (0.99 ± 0.21, n = 4), GluN2B-K79AzF (1.07 ± 0.13, n = 4), GluN2B-T83AzF (0.97 ± 0.10, n = 9), GluN2B-Q110AzF (1.29 ± 0.28, n = 3), GluN2B-I111AzF (1.21 ± 0.05, n = 2), GluN2B-Q118AzF (1.11 ± 0.18, n = 5) (Fig. 2b, Supplementary Fig. 2b). In contrast, wild-type receptors were almost unaffected (1.08 ± 0.12, n = 26), confirming that the UV treatment *per se* does not cause significant functional changes or photo damage. Taken together, these results indicate that potentiation in GluN1/GluN2B-I82AzF and GluN1/GluN2B-F114AzF receptors are mediated through AzF photo-chemistry.

UV induced relative currents were also measured for GluN2A AzF mutants, (Fig. 2c,d, Supplementary Figs 1b,d and 2a). Strikingly, none of the eight mutants were UV sensitive (GluN2A-wt: 1.18 ± 0.12, n = 11; GluN2A-P79AzF: 1.26 ± 0.11, n = 3; GluN2A-K80AzF: 1.17 ± 0.03, n = 3; GluN2A-I83AzF: 1.00 ± 0.02, n = 2; GluN2A-T84AzF: 1.23 ± 0.11, n = 13; GluN2A-Q111AzF: 1.17 ± 0.12, n = 4; GluN2A-M112AzF: 1.20 ± 0.05, n = 2; GluN2A-F115AzF: 1.11 ± 0.11, n = 7; GluN2A-Q119AzF: 1.29 ± 0.003, n = 2). These results are consistent with a previous finding that the GluN2B NTD has specific functional features which are lacking in GluN2A NTD^14^. To rule out the possibility of amber suppression leakage in GluN2A experiments, oocytes were incubated in the absence of AzF (Supplementary Fig. 1b). All mutants had minimal read-through in the absence of AzF.

### Light dependent potentiation of a selected GluN2B mutant

**Figure 3:**
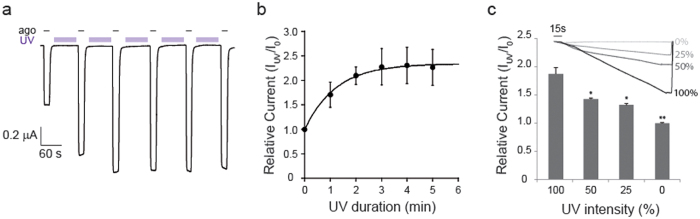
Light-dependent potentiation of GluN1/GluN2B-F114AzF. (**a**) A representative current trace at −60 mV shows functional effect under five light pulses (1 min each, 365 nm, 42 mW/cm^2^) applied sequentially. (**b**) Bar plot summarizing data presented in (**a**) shows relative currents (I_uv_/I_0_) measured at −60 mV as a function of different UV treatment durations: 1 min (1.71 ± 0.26, n = 9), 2 min (2.10 ± 0.18, n = 8), 3 min (2.28 ± 0.37, n = 8), 4 min (2.31 ± 0.37, n = 8), 5 min (2.27 ± 0.37, n = 6). (**c**) Bar plot of relative currents (I_uv_/I_0_) measured as a function UV intensity: 100% (1.84 ± 0.11, n = 3), 50% (1.42 ± 0.02, n = 2), 25% (1.32 ± 0.03, n = 2), 0% (1.00 ± 0.01, n = 3). (*Inset*) Normalized current traces measured using different UV intensities.

To evaluate the kinetics of light-dependent potentiation, we examined the relationship between light power and receptor functional changes of the GluN1/GluN2B-F114AzF receptors due to its strongest UV response. We first analyzed the kinetic response by modulating the duration of light exposure. To circumvent progressive long exposure of agonists, we applied a protocol in which agonist-induced currents are interspersed by UV illumination in the agonist-free (inactive) condition, based on the observation that similar UV potentiation effect can be produced in the resting state (Supplementary Fig. 3). In this protocol, we have applied the UV illumination for duration of one-minute in the resting state, and then co-agonists were applied (Fig. 3a). Plot of relative current versus cumulative illumination time (i.e. time spent under UV) reveals that the potentiation effect reaches to a plateau after the third one-minute UV treatment (Fig. 3b).

We then investigated the relationship between the light power and current potentiation by modulating UV intensities. Using a 42 mW/cm^2^ LED light source, 100% UV illumination evoked a 1.84 ± 0.11 (n = 3) relative current after three minutes of illumination (Fig. 3c). Decreasing the UV intensity (%) led to progressively smaller relative current (50% UV illumination: 1.42 ± 0.02, n = 2; 25% UV illumination: 1.32 ± 0.03, n = 2). No significant change in current amplitude was measured with 0% intensity (1.00 ± 0.01, n = 3). These results demonstrate that the energy of light directly controls the kinetics of receptor potentiation. Light induced receptor potentiation occurs both in the active and resting state, and has reaction kinetics on the minute time scale (τ_on_ = 49.5 s).

### Allosteric potentiation through inter-subunit photo-cross-linking

**Figure 4:**
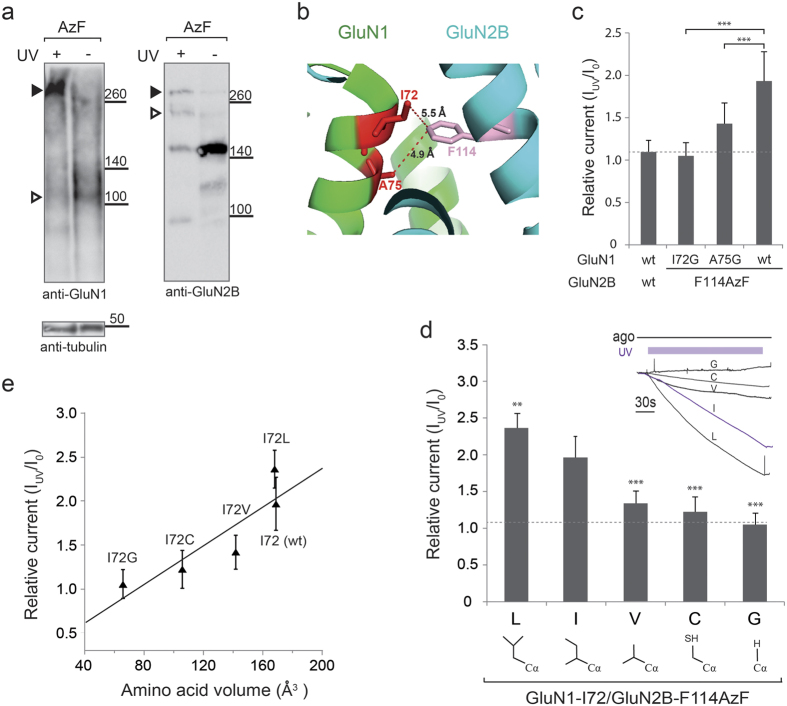
Heterodimer formation of GluN1/GluN2B-F114AzF after UV treatment. (**a**) Immunoblots from *Xenopus* oocytes expressing either F114AzF mutant or wt receptors. Oocytes were either treated with (+) or without (−) UV. Samples were analyzed by anti-GluN1 and anti-GluN2B antibodies. GluN2B monomer runs at ~180 kDa (empty triangle), GluN1 monomer runs at ~110 kDa (empty triangle), and GluN1/GluN2 heterodimer runs at ~290 kDa (indicated by a solid triangle). Non-injected oocytes (n.i.) served as a blank control. (**b**) Local environment around the GluN2B-F114 site in the GluN1/GluN2B NTD dimer. The potential candidates on the GluN1 for the UV cross-linking to F114 site are highlighted. Distances are indicated in Å. (**c**) Relative currents of wt receptors and GluN2B-F114AzF paring with GluN1 glycine mutants (I72G, A75G) and wt GluN1 receptors. Values are 1.05 ± 0.16, 1.43 ± 0.25, 1.97 ± 0.29, n = 4–21, respectively. (**d**) Changes in current amplitude after UV illumination on GluN1wt/GluN2B-F114AzF or receptors incorporating an additional substitution (L, V, C, G) at position GluN1-I72. Values are: 2.36 ± 0.2, 1.32 ± 0.18, 1.22 ± 0.20, 1.05 ± 0.16, n = 5–8. Dashed line indicates the UV induced relative current for the wild-type. (*Inset*) Normalized current traces for GluN1-I72 and its substitutions. (**e**) The GluN1-I72 residue volume and the UV-induced current potentiation are strongly correlated (linear regression, y = 0.18 + 0.01x, R = 0.89).

In order to determine the heterodimer formation between subunits of GluN1 and GluN2B-F114AzF through photo-cross-linking, we performed western-blotting experiments (Fig. 4a, Supplementary Fig. 4). To facilitate an immunoprecipitation of NMDARs from cell lysates, we have implemented an HA-tagged GluN1 (HA-GluN1), which was made by fusing a 9 amino acid HA-tag on the N-terminus of GluN1. To validate if this construct has the same UV potentiation effect as the wild-type GluN1, we have performed the UV functional assay (Supplementary Fig. 5), demonstrating no obvious difference between HA-GluN1/GluN2B-F114AzF and GluN1/GluN2B-F114AzF. Western blotting analysis of oocyte lysates after HA immunoprecipitation, amber mutant GluN2B rescued by AzF could be detected as a characteristic 180 kDa band (Fig. 4a). Upon UV exposure, a band corresponding to a heterodimer of GluN1 and GluN2B subunits (290 kDa) appeared, provided the same sample loadings using tubulin as an internal control. In the absence of UV light, there is no obvious heterodimer formation as revealed by the anti-GluN1 antibody (right panel), but predominantly the GluN1 monomer band at 110kD (empty triangle). For the anti-GluN2B detection, there was a clear heterodimer band (solid triangle) for the condition of UV treated sample, whereas no obvious heterodimer band detected in the –UV condition. Since the HA-tag is on the GluN1, we expected that only the GluN2B subunit that is crosslinked to the GluN1 could be pulled-down and detected. We observed that some GluN2B monomer were present in the IP samples and can be detected by the available anti-GluN2B antibody. UV induced heterodimer bands were also detected using cell lysates of GluN1/GluN2B-F114AzF (Supplementary Fig. 4), although at a much lower intensity than the western blotting after HA-tag immunoprecipitation.

To reveal the cross-linking partner on the opposite GluN1 subunit, we combined the classical mutagenesis with the UV functional assay. Upon UV excitation, AzF usually generates a nitrene radical and forms a covalent linkage with a nearby atom at a distance of 3–6 Å^20^,^21^,^22^. Based on the GluN1/GluN2B full-length crystal structures^8^,^9^ we identified two residues in GluN1: GluN1-I72 and GluN1-A75, both situated in the α2 helix of UL and satisfying the distance criteria (Fig. 4b). We mutated these two sites individually to a glycine, which has no side chain. When paring with the GluN2B-F114AzF, we observed a significant loss of UV-induced current potentiation, whereas for the GluN1-A75G mutant, the potentiation was only partially decreased (1.43 ± 0.25, n = 16). For the GluN1-I72G mutant, the potentiation was completely abolished (1.05 ± 0.16, n = 5) (Fig. 4c). To explore the possible role of GluN1-I72 in photo–cross-linking, we then generated a series of substitutions at this position: GluN1-I72C, GluN1-I72V and GluN1-I72L (Fig. 4d). When paring with GluN2B-F114AzF, among all double mutants, only GluN1-I72G/GluN2B-F114AzF completely abolished the UV potentiation effect, whereas the GluN1-I72L/GluN2B-F114AzF slightly increases the potentiation (2.36 ± 0.20, n = 6). The GluN1-I72V and GluN1-I72C mutants partially decreased the UV potentiation (1.32 ± 0.18, n = 8; 1.22 ± 0.20, n = 5). Plotting UV-induced potentiation versus amino acid volume^23^,^24^, it demonstrated that these two parameters were remarkably correlated, with decreasing side chain size at GluN1-I72 systematically reducing the UV-mediated effect (Fig. 4d,e). The clear correlation between the distance changes in AzF-mediated crosslinking suggests GluN1-I72 as a plausible cross-linking partner. The photo-cross-linking between AzF and GluN1-I72 possibly “locks” the GluN1/GluN2B UL-UL interface, thus affecting the gating of the receptor.

**Figure 5:**
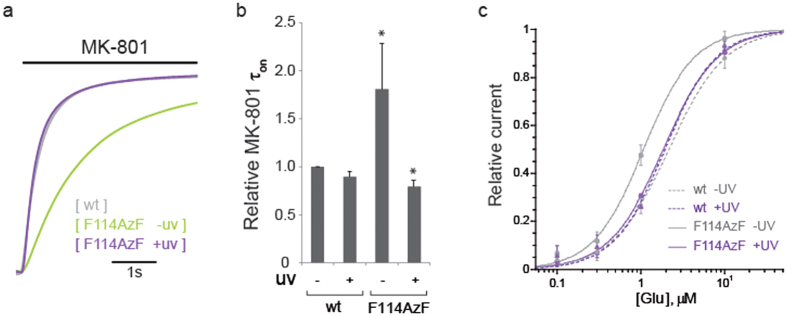
UV induced photo-cross-linking changes open probability (P_o_) and glutamate affinity. (**a**) Comparison of the inhibition kinetics in 50 nM MK-801 of wild-type (grey) and mutant receptors GluN1/GluN2B-F114AzF before (green) and after (violet) UV illumination. Current traces were normalized (derived by fitting with a single component function). (**b**) Relative MK-801 *τ*_on_ values are: wild-type without UV (1.00, n = 3); wild-type with UV (0.90 ± 0.06, n = 3); GluN1/GluN2B-F114AzF without UV (1.81 ± 0.47, n = 4); with UV (0.80 ± 0.07, n = 3). Error bars represent the standard deviation. (**c**) Glutamate dose-response curves for wt GluN1/GluN2B receptors before (EC_50_ = 2.15 ± 0.14 μM, nH = 1.29) and after UV (EC_50_ = 1.93 ± 0.29 μM, nH = 1.43); for GluN1/GluN2B-F114AzF receptors before (EC_50_ = 1.08 ± 0.08 μM, nH = 1.45) and after (EC_50_ = 1.85 ± 0.07 μM, nH = 1.35) UV treatment. n = 3–7 for each group.

To analyze the gating property change, we assessed channel maximal open probability (P_o_) using MK-801, a specific open-pore blocker of NMDARs^25^. Because the rate at which MK-801 inhibits the macroscopic NMDAR responses is proportional to the level of channel activity (i.e. P_o_), this MK-801 assay is classically used to index NMDAR channel activity. Surprisingly, before UV treatment, MK-801 inhibition kinetics of the mutant was significantly slowed down compared to wild-type receptors, indicating a marked decrease of P_o_ (Fig. 5a,b). After UV treatment, MK-801 inhibition kinetics was restored to the wild-type level. Therefore, the light induced potentiation is a dynamic transition from low P_o_ to the wild-type P_o_ process.

To determine if the P_o_ change is related to the agonist affinity change, we quantified EC_50_ values for the glutamate before and after UV light (Fig. 5c). Without UV treatment, there is a moderate two-fold increase of 1.08 ± 0.08 μM for the mutant receptors, compared with 2.15 ± 0.14 μM of the wild-type (Fig. 5c). After UV treatment, the glutamate-binding curve for the mutant receptors was superimposed with the wild-type. The mutant and wild-type receptors before and after UV light all reached a saturating current at 100 μM (in which we have assessed the P_o_), suggesting the moderate change in glutamate affinity has no influence on the P_o_ of the mutant.

### Constraining NTD interfacial flexibility reduces transduction of ifenprodil inhibition

**Figure 6:**
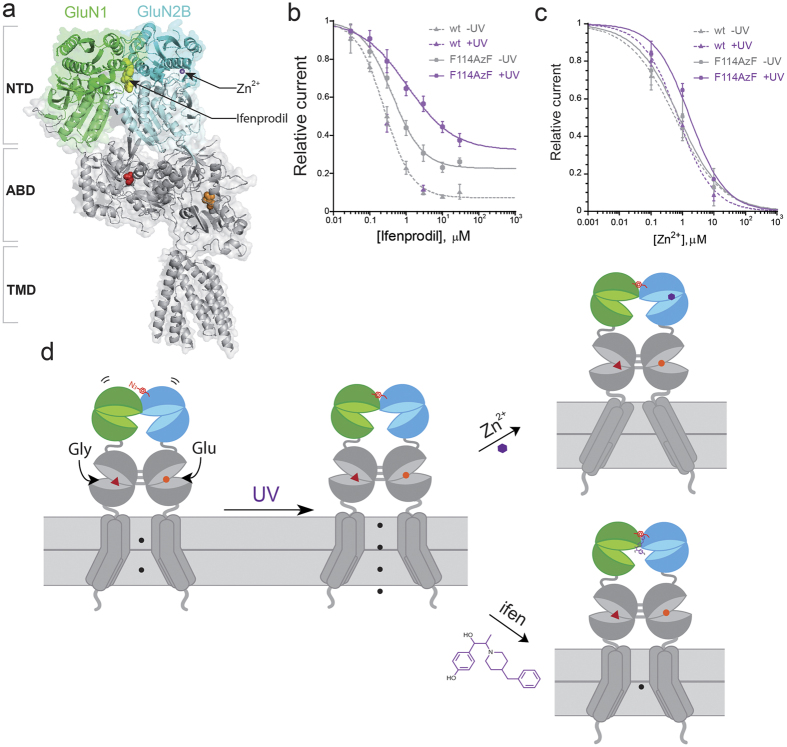
Impact of photo-cross-linking on pharmacological properties. (**a**) Side view of the crystal structure of GluN1/GluN2B heterodimer (PDB 4PE5). GluN1/GluN2B receptors harbor multiple binding sites for extracellular small-molecule ligands acting as subunit-selective allosteric modulators. Binding pockets of two allosteric inhibitors ifenprodil and Zn^2+^ are indicated. Ifenprodil (yellow sphere, PDB 4PE5) sits at the interface of two subunits and Zn^2+^ (grey sphere, PDB 3JPY) binds at the GluN2B NTD cleft. Co-agonists Gly (red) and Glu (organge) are represented as spheres. (**b**) Ifenprodil sensitivity of wt and GluN2B-F114AzF receptors before and after UV treatment. IC_50_ (μM), Hill coefficients (nH), maximal inhibition values are, respectively: 0.25 ± 0.02, 1.14, 0.93 for wt receptors; 0.41 ± 0.05, 1.04, 0.77 for GluN2B-F114AzF before UV; and 1.05 ± 0.25, 0.69, 0.67 after UV. n = 3–8 for each series. (**c**) Zn^2+^ dose-response curves for wt GluN1/GluN2B receptors before (IC_50_ = 0.58 ± 0.13 μM, nH = 0.59) and after UV (IC_50_ = 0.72 ± 0.12 μM, nH = 0.78); for GluN1/GluN2B-F114AzF receptors before (IC_50_ = 0.78 ± 0.25μM, nH = 0.62) and after (IC_50_ = 1.72 ± 0.60 μM, nH = 0.77) UV treatment. n = 3–6 for each group. Before UV, no significant changes of IC_50_ for mutant compared with wt. (*p* > 0.05) (**d**) Proposed model for light induced allosteric modulation of GluN1/GluN2B-F114AzF receptors. Shown are two GluN1 subunits (green) and GluN2B subunits (blue) forming a functional tetramer. Molecule AzF is highlighted in red. Agonists binding (glycine as a red dot and glutamate as an orange triangle) lead to opening of the ion channel. UV illumination induces AzF crosslinking to GluN1, which constrains the NTD flexibility and induces NTD conformational changes that leads to channel potentiation. After UV crosslinking, Zn^2+^ binding (purple dot) at the GluN2B NTD leads to the full inhibition. Ifenprodil binding at the NTD UL-UL interface leads to partial inhibition.

Ifenprodil and Zn^2+^ bind at spatially distinct locations at the GluN1/GluN2B NTDs (Fig. 6a). To explore the impact of the GluN1 and GluN2B NTD interface to allosteric regulation of ifenprodil, we determined the ifenprodil sensitivities before and after UV induced crosslinking with the GluN1/GluN2B-F114AzF receptors. Remarkably, without UV treatment, we found that ifenprodil binding had a drastic change in maximal inhibition (from 93% for wild-type to 77% for mutant receptors) (Fig. 6b, Supplementary Fig. 6a, Supplementary Table 1). After UV treatment, the maximal inhibition was further decreased to 67% for the mutant receptors. Meanwhile, IC_50_ of ifenprodil was only moderately decreased (0.41 ± 0.05 μM before UV and 1.05 ± 0.25 μM after UV for the mutant, 0.25 ± 0.02 μM for wt), indicating that AzF and crosslinking had no significant influence on ifenprodil binding. In addition, it suggests that the significant decrease in the ifenprodil maximal inhibition for the mutant before and after UV is not due to the change of ifenprodil binding, but rather an intrinsic functional feature of the interface.

To further understand the physiological relevance of the dimer interface on the Zn^2+^ inhibition, we have performed Zn^2+^ titrations before and after UV treatment on the GluN1/GluN2B-F114AzF receptors. IC_50_ was slightly increased to 1.72 ± 0.60 μM for the UV treated mutant receptors, compared to 0.72 ± 0.12 μM of the wild-type (Fig. 6c, Supplementary Fig. 6b). Before UV treatment, IC_50_ had no significant change (P = 0.22, Student’s t-test) indicating the interface has minimal effect on the Zn^2+^ binding. In addition, there was no change in the maximal inhibition. Maximal inhibition is associated with the transduction of the allosteric modulation to gating. Our comparisons between ifenprodil and Zn^2+^ affinities using the light-sensitive mutant suggest the transduction of ifenprodil inhibition, but not Zn^2+^, is reduced (Fig. 6d). Since UV treatment creates a covalent crosslinking bond at UL-UL interface between GluN1 and GluN2B NTD, our results reveal that this interface plays a specific functional role in the transduction of ifenprodil inhibition.

### The impact of allosteric modulations on the NTD interfacial arrangement

**Figure 7:**
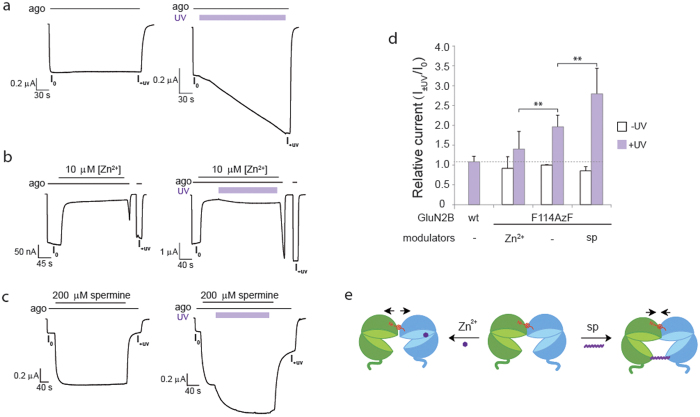
UV sensitivity of GluN1/GluN2B-F114AzF in the presence of allosteric modulators. (**a**) Representative current traces showing UV induced current potentiation of GluN1/GluN2B-F114AzF in the presence of co-agonists with or without UV. (**b**) Representative current traces showing GluN1/GluN2B-F114AzF receptors measured in the presence of Zn^2+^ (10 uM) with or without UV. (**c**) Representative current traces showing GluN1/GluN2B-F114AzF receptors measured in the presence of spermine (200 μM) with or without UV. (**d**) Relative currents measured in different conditions in the presence (violet bars) or absence (white bars) of UV: Zn^2+^ (−UV: 0.92 ± 0.29, n = 7; +UV: 1.40 ± 0.43, n = 10); “−” (in the presence of co-agonists, −UV: 1.00 ± 0.01, n = 3; +UV: 1.97 ± 0.29, n = 21); and spermine (−UV: 0.85 ± 0.10, n = 4; +UV: 2.80 ± 0.64, n = 11). (**e**) Schematic of NTD dimer rearrangement through allosteric modulations. AzF is highlighted in red. Left panel: Zn^2+^ (purple dot) binding at the GluN2B-NTD subtly separates the interface. Right panel: Spermine (purple wave) binding between the UL-UL of NTDs reduces the gap at the interface to favor the AzF crosslinking.

To understand how the NTD dimer interfacial contact influences allosteric modulation, we have quantified UV-induced potentiation in the presence of different modulators, including a GluN2B specific positive allosteric modulator spermine^26^. In the absence of modulators, the current induced by co-agonists remains constant (Fig. 7a, left panel), and potentiation is observed with the UV stimulation (Fig. 7a, right panel). In the presence of co-agonists and 10 μM Zn^2+^, activation is rapidly inhibited due to receptors entering into a desensitized state^27^ (Fig. 7b, left panel), which can be re-activated upon washing off the Zn^2+^. In the presence of UV light (Fig. 7b, right panel), the full agonist-induced current was measured again upon washing off the Zn^2+^, yielding the value of I_+uv_. The relative current I_+uv_/I_0_ is calculated (1.40 ± 0.43; n = 10) and compared to the relative current observed without UV (0.92 ± 0.29; n = 7) (Fig. 7d). Surprisingly, in the presence of ifenprodil, the potentiation effect was completely abolished (Supplementary Fig. 7a,b). One explanation is that ifenprodil binding, causes a slight separation of the F114AzF from the GluN1-I72, which disables the AzF crosslinking (Supplementary Fig. 7c,d).

In the presence of 200 μM spermine at pH 6.5, the activation of receptors is rapidly potentiated and reaches to a 4.6 fold increase, which could be washed back to the original agonists level (Fig. 7c, left panel). In the presence of UV light, we noticed that there was a strong potentiation added onto the spermine potentiation (Fig. 7c, right panel, 7d, 2.80 ± 0.64, n = 11; 0.85 ± 0.10, n = 4). Comparing all I_+uv_/I_0_ values (Fig. 7d,e), it is noticeable that Zn^2+^ reduces the UV potentiation while spermine enhances it. In addition, this UV induced additional potentiation in the presence of spermine follows the same kinetics (τ_on_ = 50.8 s) as measured in the agonists alone (τ_on_ = 49.5 s). Taken together, our results reveal two mechanisms: (1) Functional changes induced by light and allosteric modulations are independent and not mutually exclusive; (2) Since AzF mediated crosslinking is strictly distance dependence^14^,^20^,^21^,^22^, it implies that the presence of allosteric modulators can tweak the interfacial contact, with Zn^2+^ subtly separating the interface while spermine enhancing it by favoring crosslinking geometry (Fig. 7e).

## Discussion

Recent progress^3^,^8^,^9^ in structural biology of NMDARs has begun to make it possible to differentiate various forms of allosteric modulations on a structural basis. Up to now, two full-length structures^8^,^9^ and one isolated NTD dimer structure^10^ (all crystallized with ifenprodil and derivatives), and a recent x-ray crystal structure of apo NTD dimer structure are available^3^. They show conserved interfacial contacts between GluN1 and GluN2B at the NTD dimer upper lobe – upper lobe (UL-UL) interface. It raises the question whether this interface has a functional role. The light-induced trapping of GluN1/GluN2B heterodimer in the absence of ifenprodil reveals a close contact between GluN2B-F114 and the neighboring GluN1 subunit with or without agonists, suggesting this interface is present in the resting and activated state. The differential binding profiles of ifenprodil and Zn^2+^ on the light-sensitive GluN1/GluN2B-F114AzF mutant reveals a functional element unique to the ifenprodil transduction. Our results strengthen strategies for developing allosteric small-molecules or antibodies targeting the GluN1/GluN2B NTD interface as subtype-selective therapeutic agents. The GluN1/GluN2B-F114AzF mutant is ideal for the mechanistic characterization of binding profiles of newly developed ifenprodil derivatives which are promising therapeutic agents in neuropharmacology^7^,^13^.

The UV potentiation effect is remarkable, because it drastically contrasts the inhibition effect of ifenprodil binding. It provides the first evidence for a possible mechanism to positively regulate the receptor function at the ifenprodil binding interface, which is a dynamic transition from low P_o_ to the restoration of the WT-like P_o_ due to the UV induced interfacial crosslinking. Taken together with the our previously identified light inhibiting mutant GluN1-Y109AzF/GluN2B, we for the first time reveal NTD rearrangements at this interface can bidirectionally modulate receptor function. The kinetics of NTD-crosslinking induced potentiation is on the order of minute (τ_on_ = 49.5 s), which is slightly faster than our previously identified inhibiting AzF mutant (τ_on_ = 80 s)^14^. Compared to all reported light-sensitive receptors and ion-channels using different Uaas targeting different domains: (1) Bpa inserted at the agonist binding domain (ABD) dimer interface of AMPARs (τ_on_ = 3–5.6 s)^28^; (2) caged-serine at the pore region of voltage-gated potassium channel Kir2.1 (τ_on_ = 0.3 s)^29^; and (3) caged-tyrosine at the intracellular domain of nAChR (τ_on_ = 0.1–8 ms)^30^, the kinetics of NMDAR-AzF mutants are significantly slower. Such difference may likely represent NTD mediated conformational switches specific to the NMDARs, although systematic comparisons using the same light-source may provide insight in the differential regulatory mechanisms.

Our photochemical study of the heterodimer interface extended previous works directed at this allosteric interface using classical fluorescent-labeling^31^,^32^ and disulfide-crosslinking approaches^10^,^33^ which could not reveal any functional role. Compared to disulfide crosslinking studies at this dimer interface, photo-cross-linking by AzF only requires one mutation in one subunit, while double-cysteine screening requires two modifications at the interface leading to many different combinations and could change the function of the receptors. Compared to the LRET methodology^31^,^32^ relying on fluorescent probes showing no distance changes at this interface, our detection is sensitive due to the distance dependence of the photo-cross-linking probe which could occur only at a 3–6 Å scale. In addition, UV potentiation modulated by the GluN1-I72 residue (side-chain systemically shortened by the conventional site-directed mutagenesis) reveals how distance changes at the interface affect allostery. Between two UV crosslinking Uaas (AzF and *p*-benzoyl-L-phenylalanine, Bpa), we have found that when inserting Bpa which is bulkier than AzF (Supplementary Fig. 8), no light induced functional changes was observed. This is not surprising because using conventional site-directed mutagenesis on the GluN2B-F114 site, ifenprofil sensitivity had drastic changes^10^. Remarkably, GluN2B-F114AzF mutation and photo-cross-linking only slightly decreased the ifenprodil sensitivity, demonstrating the advantage of AzF being the non-perturbing probe to detect the protein functional change in NMDARs^14^. Although in AMPARs, Bpa had a clear advantage over AzF in detecting desensitization at the ABD interface^28^. Taken together, the approach to introduce light-sensitive Uaas provide a unique approach to identify subtle structural changes.

Our success relies on the heterologous expression system - *Xenopus laevis* oocytes – a robust expression vehicle for the efficient genetic code expansion and functional analysis of LGICs^17^,^34^,^35^,^36^,^37^,^38^. The expression level is particularly critical for NMDARs, which requires at least two different subunits to assemble functional receptors. Oocyte system is appealing due to its high protein expression level of the heteromeric LGICs^35^,^36^,^37^. In addition, our convenient procedure of Uaa incorporation in oocytes enables the efficient expression of functional NMDAR mutants^17^. The robustness of the oocytes enables us to compare different conformation states before and after light on the same cell, eliminating cell-to-cell variation. In recent demonstrations of light-sensitive Kir2.1^29^ and AMPARs^28^, functional analyses using the patch-clamp technique in HEK293 mammalian cells were applied. The technical difficulty compared to the TEVC and the lower light-induced responses^14^ in mammalian cells make oocytes more appealing for structural function studies of LGICs^37^,^38^.

Complement to opto-neuropharmaocological approaches, which relies on introducing photo-sensitive ligands to engineer light-sensitive iGluRs^39^, the genetic code expansion directly inserts the light-sensitive moiety into the protein at any allowed site. In recent years, the genetic code expansion has rapid developments by implementing the pyrrolysyl-tRNA synthetase/tRNA pair^40^. The major advantage of the pryrolysyl-tRNA synthetase is its high substrate side-chain promiscuity, which leads to the successful encoding of Uaas with other light-functionalities such as the azobenzene moiety^41^ that can be reversibly switched between two conformations using different wavelength of light. Reprogramed genetic code systems can now be implemented in eukaryotic cells^42^,^43^, including neurons^14^,^29^,^44^ and whole animals^45^,^46^, making both the light sensitive receptors developed herein and photo-control of other Uaa incorporated neuronal proteins of broad applicability^14^,^28^,^29^,^30^. We envision applications of various light-sensitive Uaas in other neuronal receptors through the genetic code expansion.

## Experimental Methods

### Materials

*p*-azido-L-phenylalanine (AzF) was purchased from Chem-Impex International (Wood Dale, IL) and *p*-benzyol-L-phenylalanine from Bachem (Bubendorf, Switzerland), respectively. HEPES, l-glutamate, glycine, DTPA, spermine were obtained from Sigma–Aldrich. D-APV and (+)-MK-801 were purchased from Ascent Scientific (Bristol, UK). Ifenprodil was purchased from Synthélabo (France).

### Plasmids and site-directed mutagenesis

Plasmid pSVB.Yam carrying the gene encoding the amber suppressor tRNA was derived from *B*. stearothermophilus Tyr-tRNA_CUA_ and has been described previously^47^. The amnioacyl-tRNA synthetases (aaRS) for AzF were constructed as previously described^48^. The pcDNA3-based expression plasmids for rat GluN1–1a, rat GluN2A, and mouse GluN2B have been described previously^4^. The amber mutations were introduced into GluN2A and GluN2B by using a Quikchange site-directed mutagenesis kit (Stratagene).

### Injection of plasmid DNAs into oocytes and Uaa incubation

Oocytes were prepared and injected as described previously^17^. Recombinant NMDARs were expressed in *Xenopus laevis* oocytes after nuclear injection of 36 nl of a mixture cDNAs encoding various GluN1 and GluN2 subunits (ratio 1:1, 10 ng/μl for GluN2A and 30 ng/μl for GluN2B). For Uaa incorporation, oocytes were co-injected with a 36 nl mixture of cDNAs containing GluN1, GluN2, Yam, and aaRS as follows, unless otherwise indicated in the text: GluN1-wt (60 ng/μl), GluN2A-F115AzF (60 ng/μl), Yam (5 ng/μl), AzF-RS (2.5 ng/μl); or, GluN1-wt (80 ng/μl), GluN2B-F114AzF (80 ng/μl), Yam (10 ng/μl), AzF-RS (5 ng/μl). After injection, oocytes were incubated at 19 °C in a Barth solution (88 mM NaCl, 1 mM KCl, 0.33 mM Ca(NO_3_)_2_, 0.41 mM CaCl_2_, 0.82 mM MgSO_4_, 2.4 mM NaHCO_3_, 10 mM HEPES, pH adjusted to 7.6 with NaOH) supplemented with gentamicin sulfate (50 ng/ml) and D-APV (50 μM). AzF was dissolved with sonication in Barth solution (stock solution at 10 mM), and diluted (1 mM) for oocytes incubation.

### Electrophysiology

For all experiments, the standard external solutions contained: 100 mM NaCl, 300 mM BaCl_2_, and 5 mM HEPES, pH adjusted to 7.3 with KOH. Other than for the glutamate sensitivity assay (100 μM glycine in the presence of various concentrations of glutamate), NMDAR-mediated currents were induced by applying glutamate (100 μM) and glycine (100 μM), which activate the receptors at the maximum level. Currents were recorded and measured at a holding potential of −60 mV at room temperature.

Glutamate, ifenprodil, zinc dose response curve experiments were performed and analyzed as previously described^25^. Ifenprodil was prepared as 10 mM stock aliquots (in 1% HCl). The measurements at 30 μM were corrected by multiply with a correcting value to remove the pore blocking effect at −60 mV^49^. Zinc was prepared at 100 mM ZnCl_2_ stock (in 1% HCl). In all zero-Zn^2+^ control solutions, diethylenetriamine-pentaacetic acid (DTPA, 10 μM) was added to chelate trace Zn^2+^ and other heavy metals^25^. Spermine potentiation was analyzed as described earlier^13^. Solutions of 200 μM spermine (Sigma-Aldrich) were made by directly diluting the powder into the standard agonist solution. Spermine sensitivity was assessed at pH 6.5 in order to maximize the spermine-induced potentiation. Experiments with MK-801 were performed as described previously. MK-801 solutions (10–50 nM) were prepared by dilution of stock solution (50 μM) into agonist containing solution^4^. MK-801 time constants of inhibition (τ_on_) were obtained by fitting currents with a single-exponential component to a time window corresponding to 10–90% of maximum inhibition. Each τ_on_ was then normalized to the mean τ_on_ of wt receptors measured the same day.

### Immunoblotting

Sample preparation, non-reducing SDS-PAGE, and immunoblotting were performed as described^17^. In brief, for each condition, oocytes were cultured for three days post injection to achieve maximal protein expression (NMDAR-mediated currents >10 μA). Four oocytes from each batch was then homogenized and processed, and then separated in non-reducing conditions on SDS-PAGE gradient gels (3–8%), and dry transferred as described^17^. Immunoprecipitation of HA-tagged fusion protein is performed by incubation of the lysate with anti-HA-bound beads (anti-HA-Agarose, Sigma-Aldrich). The following antibodies were used: anti-GluN1 (1:750, mouse monoclonal MAB1586 clone R1JHL; Millipore), anti-GluN2B antibody (1:500, mouse monoclonal 75–101 clone N59/36; NeuroMab) and anti-α-tubulin antibody (1:1000, mouse monoclonal DM1A clone, Upstate). Protein bands were visualized by using secondary goat peroxidase-conjugated anti-mouse antibody (1:20,000, Jackson ImmunoResearch, West Grove, PA) with SuperSignal West Pico Chemiluminescent Substrate (Thermo Scientific).

### UV photo-cross-linking treatment

For all functional recordings in *Xenopus* oocytes, online UV light treatment with PE-2 light source (CoolLED) using a 365 nM filter channeled through optical fiber was directly applied to the dark hemisphere of the oocytes in the recording chamber. Total power measured at a distance of 200 mm from the source is 105 mW (42 mW/cm^2^), as reported by the manufacturer. For measurements in presence of agonists and allosteric modulators, the UV application duration was ~3 min. In the absence of agonist, the UV application duration was ~5 min.

For western immunoblotting, oocytes expressing wt NMDA receptors or AzF mutant receptors were transferred to a 96-well plate containing Barth solution free of AzF and agonists (one oocyte per well, animal pole facing up) on ice. Cells were irradiated for 30 min with a hand-held VL-6LC UV lamp (6 W, 365 nm, Viber Lourmat, Marne-la-Valle, France) placed on top of the plate. After UV treatment, cells were subject to western blotting analysis.

## Additional Information

**How to cite this article**: Tian, M. and Ye, S. Allosteric regulation in NMDA receptors revealed by the genetically encoded photo-cross-linkers. *Sci. Rep.* **6**, 34751; doi: 10.1038/srep34751 (2016).

## Supplementary Material

Supplementary Information
