# Supplementary material for: Allosteric regulation in NMDA receptors revealed by the genetically encoded photo-cross-linkers
Source: Sci Rep. 2016 Oct 7;6:34751. doi: 10.1038/srep34751 (PMC5054432; doi:10.1038/srep34751)
Supplement: Supplementary Information [file srep34751-s1.doc]

**Supplementary Information**

Allosteric regulations in NMDA receptors revealed by

the genetically encoded photo-cross-linkers

Meilin Tian and Shixin Ye

Correspondence should be addressed to yelehman@biologie.ens.fr

**Supplementary information includes:**

Supplementary Figures 1−8

Supplementary Table 1

**
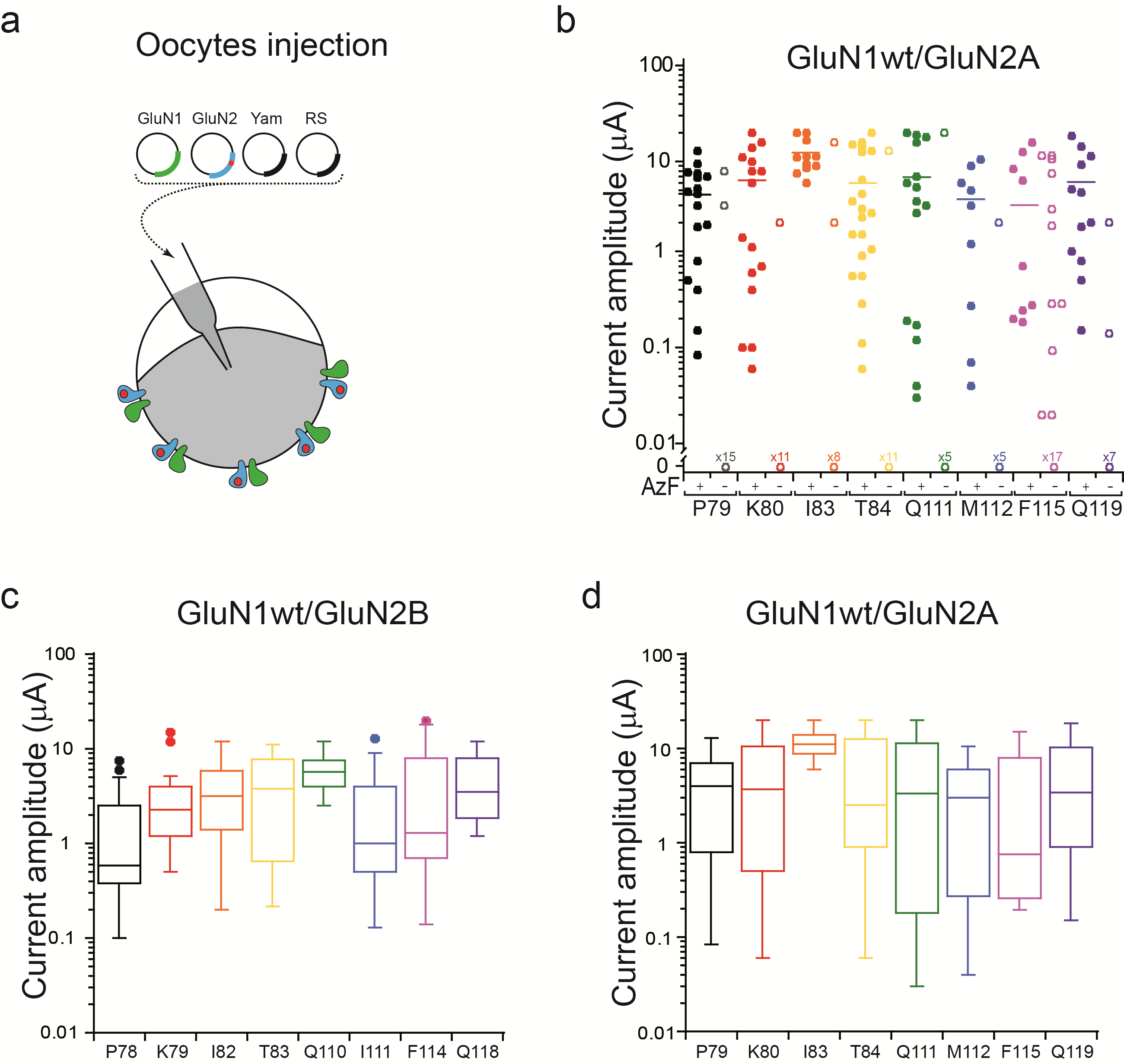
**

**Supplementary Figure 1.** **Incorporation of AzF in GluN2A receptors. a**. Schematic illustration of oocytes coinjected with four plasmids: GluN2 amber mutant, wt GluN1, Yam and AzF-RS. **b**. Currents measured from oocytes split into two batches after coinjections: one batch was incubated in the medium with 1mM AzF and one batch without AzF. For each condition, currents >10 nA were plotted and at least 5 oocytes were tested. **c,d**. Box plots of coagonists induced current responses of NMDA AzF mutants. Oocytes were injected with plasmids encoding GluN1, GluN2B (**c**) or GluN2A (**d**) with an amber stop codon mutation at the indicated site, Yam, and AzF-RS, and with AzF supplied in the medium. The bottom and top of the box are the first and third quartiles, and the band inside the box is the median. The whiskers are with maximum 1.5 IQR.

**
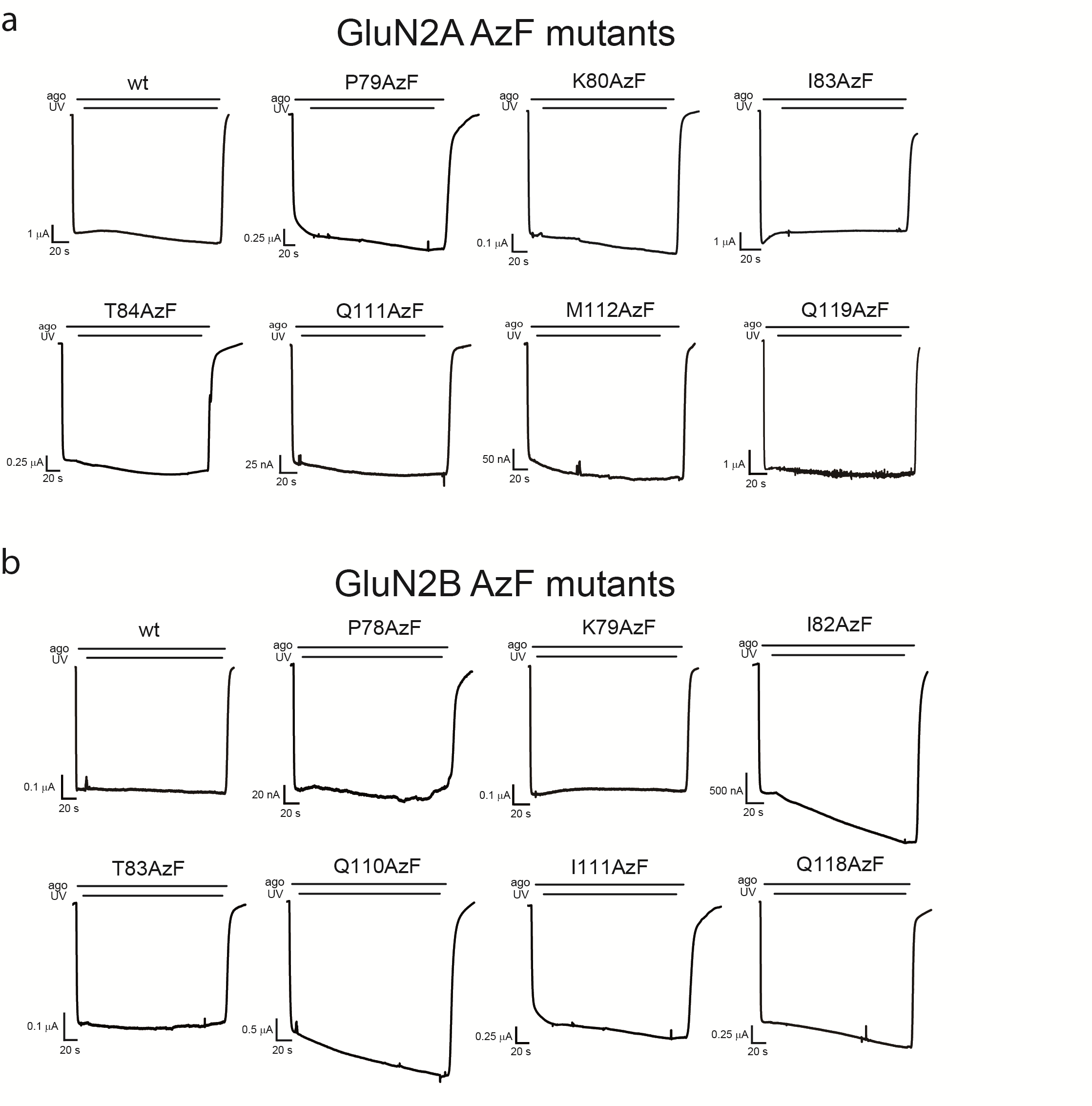
**

**Supplementary Figure 2.** **UV-sensitivity of AzF incorporated in GluN2A and GluN2B receptors.** a. Representative current traces measured from oocytes expressing wt and GluN2A AzF mutant receptors during UV illumination. **b**. Representative current traces measured from oocytes expressing wt and GluN2B AzF mutant receptors during UV illumination.

**
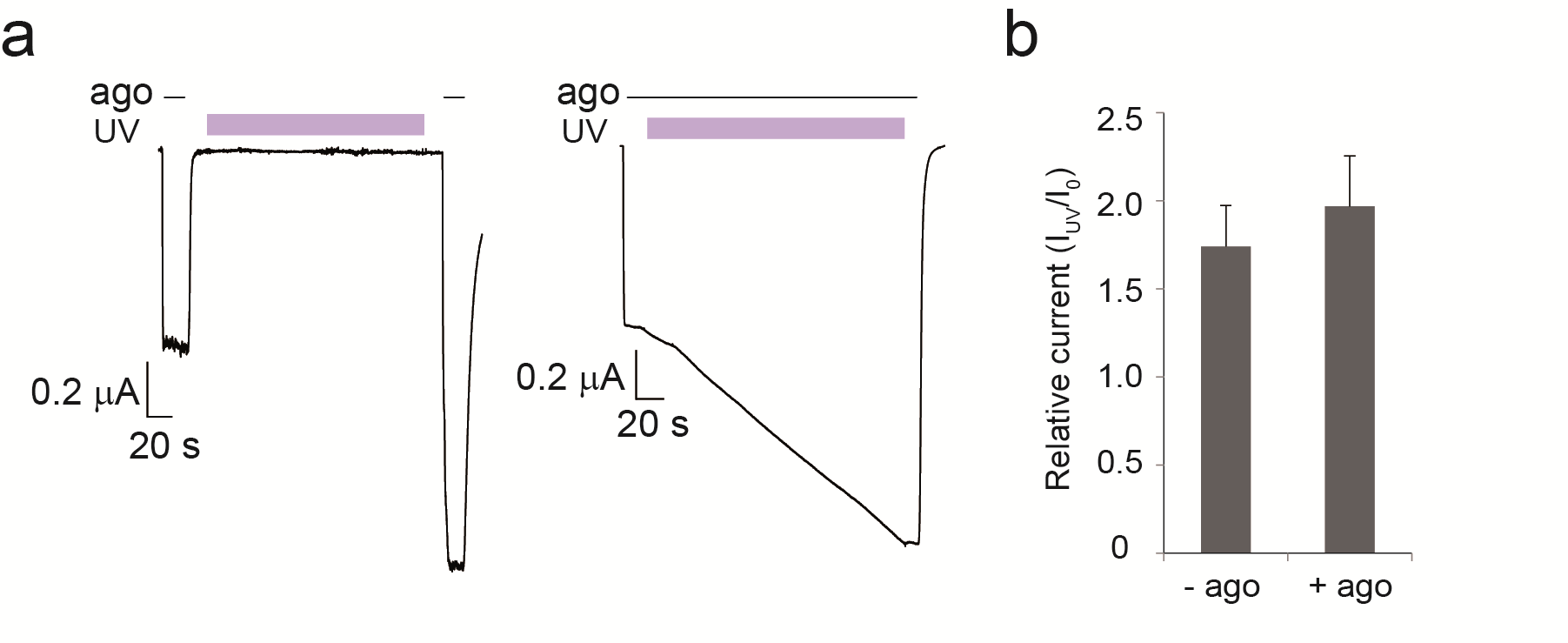
**

**Supplementary Figure 3. UV potentiation of the GluN1/GluN2B-F114AzF in resting and active states. a**. Representative current traces measured from oocytes expressing GluN1wt/GluN2B-F114AzF showed potentiation in resting (left panel) and active (right panel) state. **b**. Relative currents (I_uv_/I_0_) measured in resting (-ago: 1.74 ± 0.23, n = 3) and active (+ago: 1.97 ± 0.29, n = 21) state.

**
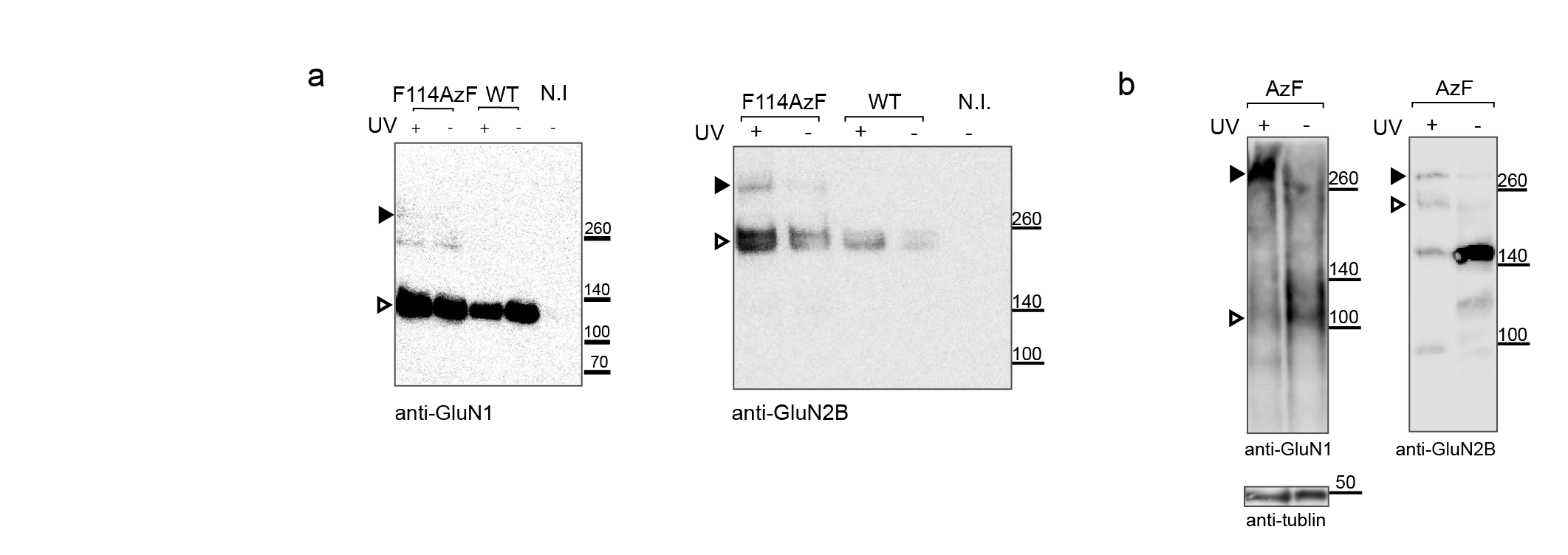
**

**Supplementary Figure 4. Immunoblots from *Xenopus* oocytes expressing either wt or GluN1wt/GluN2B-F114AzF mutant receptors.** Oocytes were either treated with (+) or without (−) UV. Cell lysates were analyzed by anti-GluN1 and anti-GluN2B antibodies. GluN1 monomer runs at ~110 kDa(empty triangle), GluN2B monomer runs at ~180kDa(empty triangle), GluN1 homodimer runs at ~220 kDa, and GluN1/GluN2B heterodimer runs at ~290 kDa (indicated by an solid triangle). Non-injected oocytes (n.i.) served as a blank control.


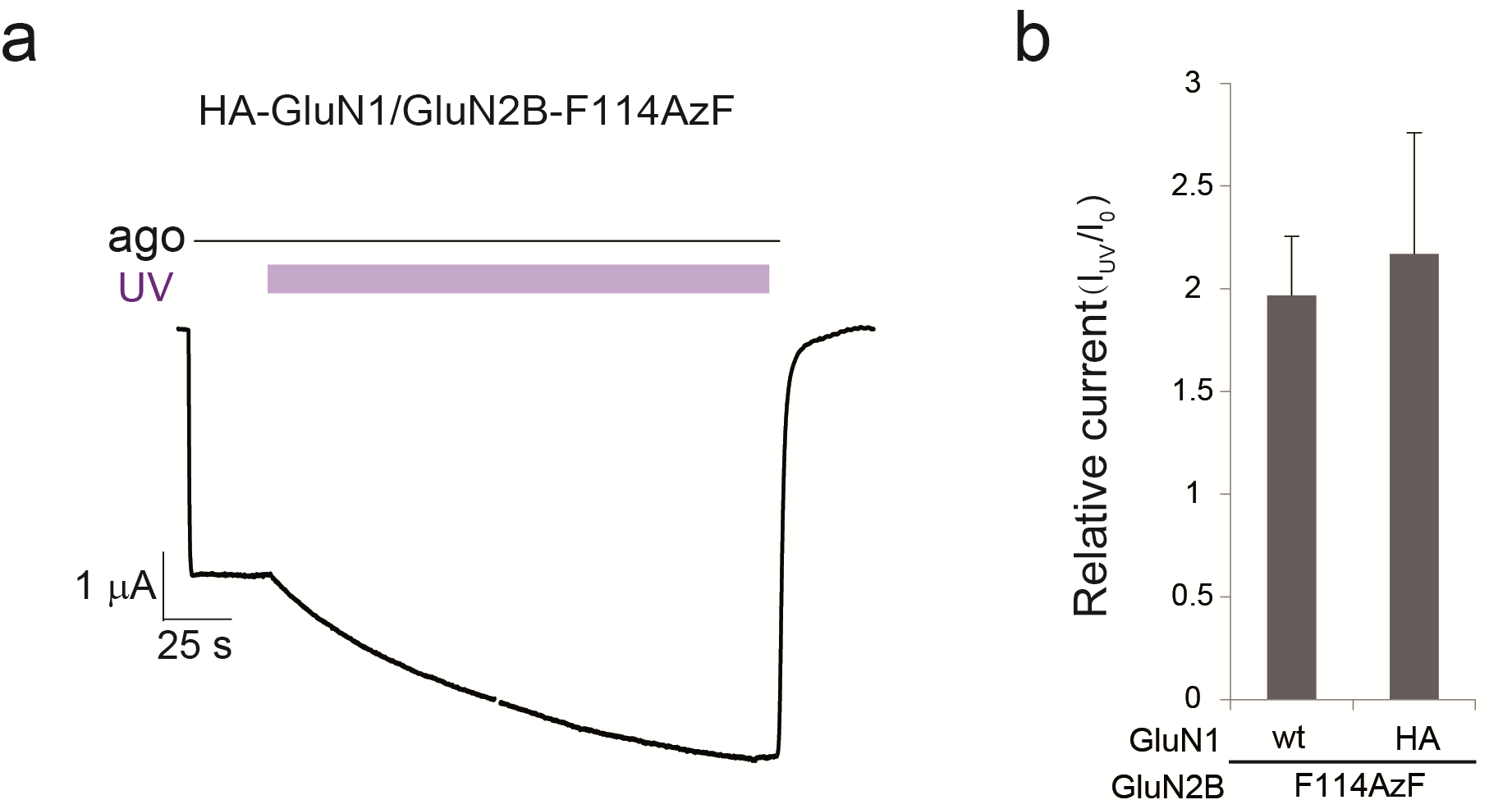


**Supplementary Figure 5.** **Light-dependent potentiation of HA-GluN1/GluN2B-F114AzF. a**. A representative current trace at -60mV shows functional effect under UV light (3min duration, 365 nm, 42mW/cm^2^) applied. **b**. Bar plot of relative currents (I_UV_/I_0_) shows there is no significant difference between HA-GluN1/GluN2B-F114AzF and wt-GluN1/GluN2B-F114AzF receptors: wt-GluN1 (1.97±0.29, n=21) and HA-GluN1 (2.17±0.59, n=2).


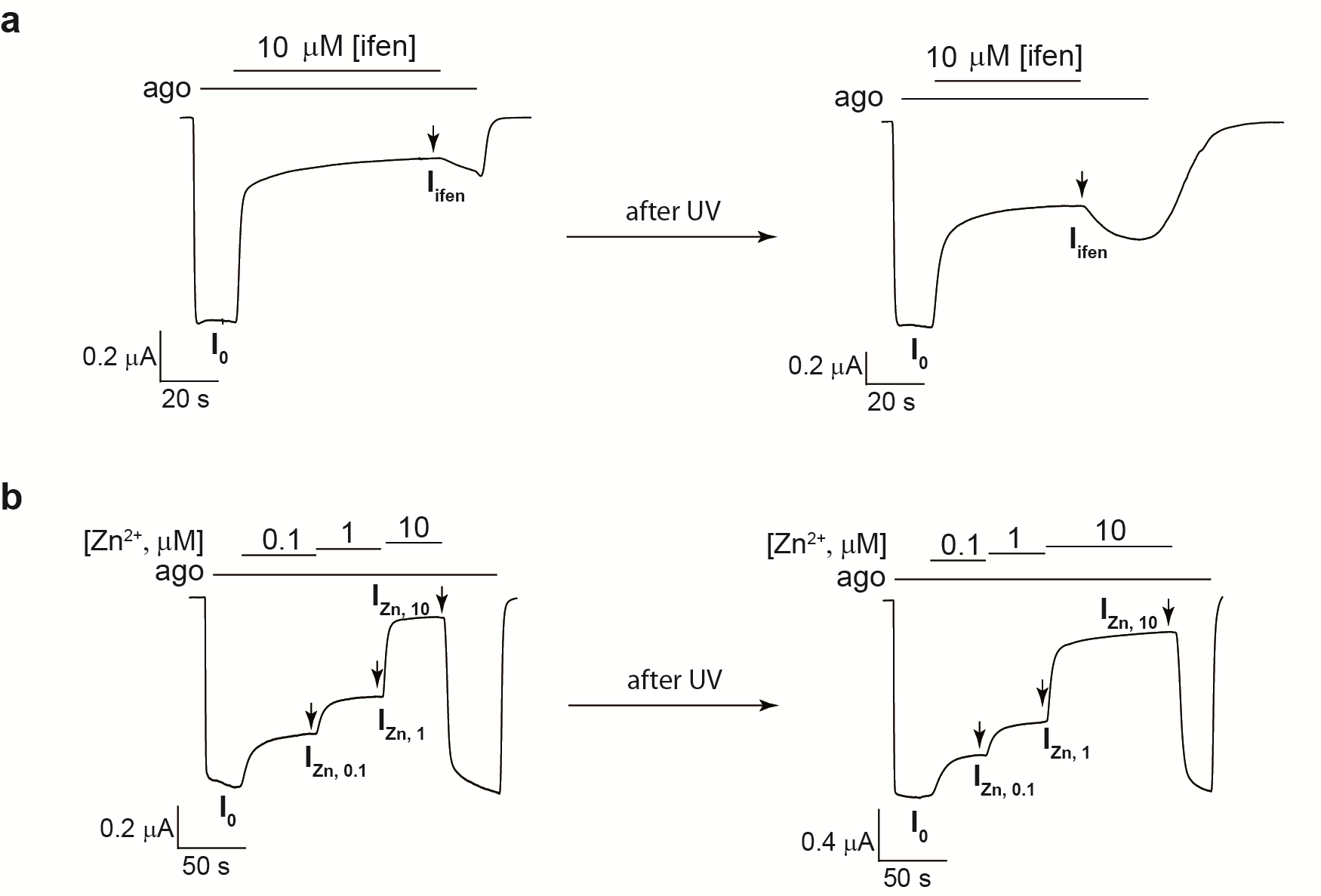


**Supplementary Figure 6.** **A comparison of the current traces obtained from oocytes expressing GluN1/GluN2B-F114AzF before and after UV treatment.** **a**. Ifenprodil was applied at 10 μM during an application of coagonists. **b**. Zn^2+^ was applied at three increasing concentrations (0.1, 1, and 10 μM) during an application of coagonists. The relative current is calculated as: I_ifen_/I_0_ at 10 μM, I_Zn,0.1_/I_0_ at 0.1 μM, I_Zn,1_/I_0_ at 1 μM, and I_Zn,10_/I_0_ at 10 μM).


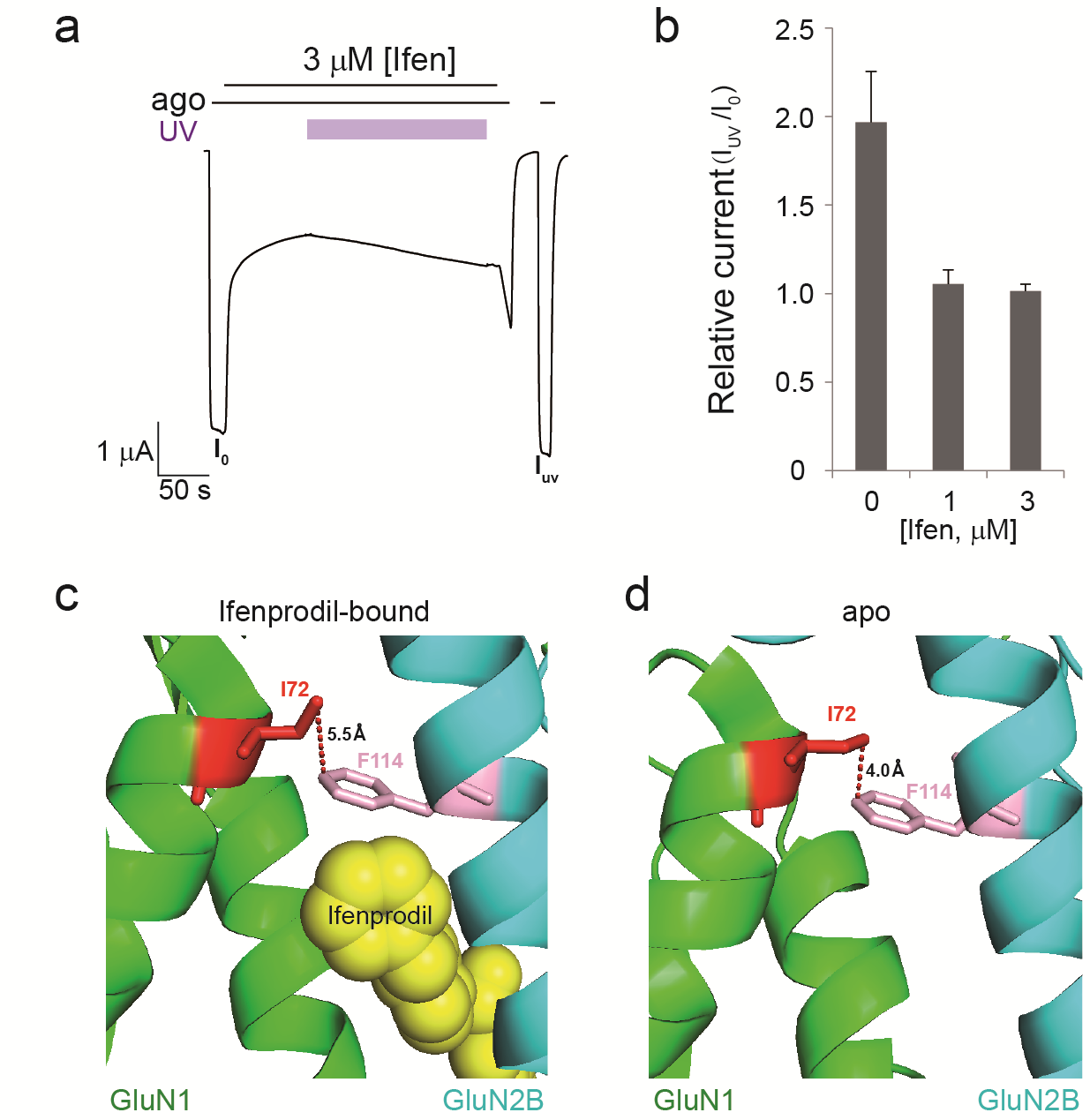


**Supplementary Figure 7.** **UV sensitivity of GluN1/GluN2B-F114AzF in the presence of ifenprodil.** **a**. A representative current trace at -60mV showing agonists induced current changes measured in the presence of 3 μM ifenprodil under UV light (3min duration, 365 nm, 42mW/cm^2^) treatment. **b**. Bar plot of relative currents (I_UV_/I_0_) measured in 0, 1, and 3 μM ifenprodil: 0 μM (1.97±0.29, n=21), 1 μM (1.05±0.08, n=3), 3 μM (1.01±0.04, n=7). **c**. Distance between GluN1-I72 and GluN2B-F114 sites in the ifenprodil- bound GluN1-GluN2B NTD structure (PDB ID, 4PE5), in comparison with the apo structure (PDB ID, 5B3J). The ifenprodil is represented by yellow spheres.


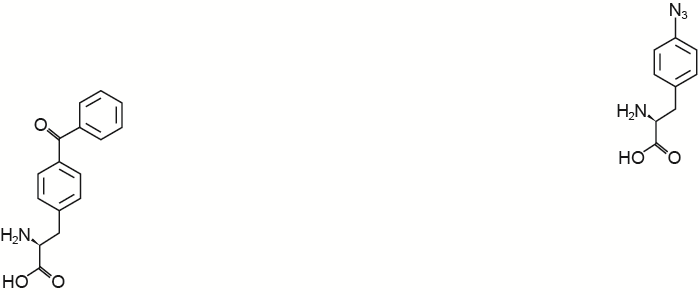
 **
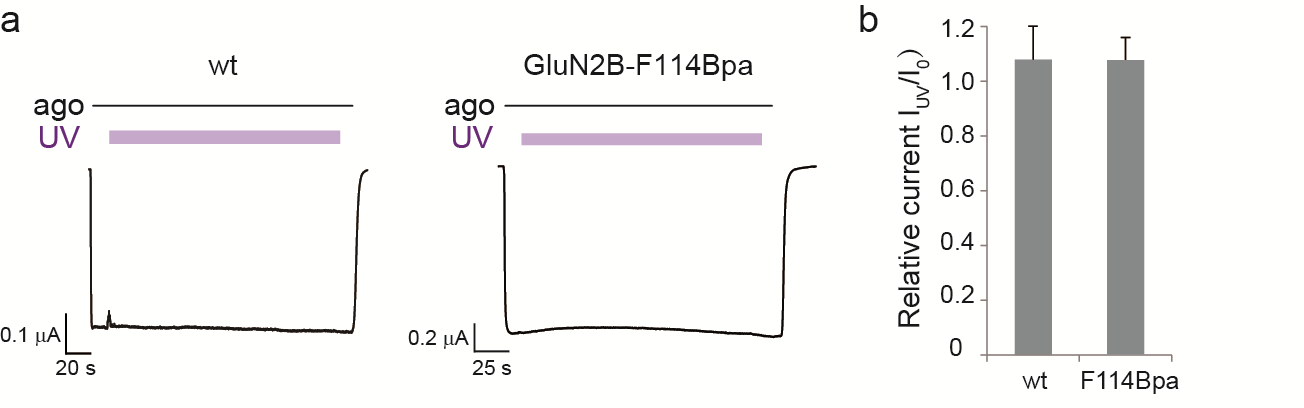
**

Bpa

**Supplementary Figure 8. UV sensitivity of GluN1/GluN2B-F114Bpa. a**. Representative current traces measured from oocytes expressing wt (left panel) and GluN2B-F114Bpa (right panel) receptors during UV illumination. **b**. Relative currents (I_uv_/I_o_): GluN2Bwt (1.08 ± 0.12, n =26), GluN2B-F114Bpa (1.07 ± 0.08, n = 5).

**Supplementary Table 1. A comparison of inhibition effects of ifenprodil and Zn^2+^ on GluN1/GluN2B-F114AzF before and after UV treatment.**

| Maximal inhibition (%) | | | | |
| --- | --- | --- | --- | --- |
|  | Ifenprodil | | Zn^2+^ | |
|  | -UV | +UV | -UV | +UV |
| 0.1uM | 16 ± 3 | 9 ± 4* | 25 ± 8 | 16 ± 7 |
| 1uM | 54 ± 4 | 35 ± 4*** | 49 ± 11 | 35 ± 4 |
| 10uM | 77 ± 3 | 55 ± 3*** | 87 ± 4 | 83 ± 1 |

*P<0.05; **P<0.01; ***P<0.001 (Student's t-test), n = 3 - 6
